# Expression and purification of functional recombinant CUL2•RBX1 from *E. coli*

**DOI:** 10.1038/s41598-021-90770-x

**Published:** 2021-05-27

**Authors:** Stephanie Diaz, Lihong Li, Kankan Wang, Xing Liu

**Affiliations:** 1 Department of Biochemistry, Purdue University, West Lafayette, IN USA; 2 Center for Plant Biology, Purdue University, West Lafayette, IN USA

**Keywords:** Biochemistry, Enzymes, Proteins

## Abstract

Cullin-2 (CUL2) based cullin-RING ligases (CRL2s) comprise a family of ubiquitin E3 ligases that exist only in multi-cellular organisms and are crucial for cellular processes such as embryogenesis and viral pathogenesis. CUL2 is the scaffold protein that binds one of the interchangeable substrate receptor modules, which consists of adaptor proteins and the substrate receptor protein. The VHL protein is a substrate receptor known to target hypoxia-inducible factor α (HIF1α) for ubiquitination and degradation. Because of its critical role in the ubiquitination of important cellular factors such as HIF1α, CRL2s have been investigated for their biological functions and the development of novel therapeutics against diseases. Given the importance of CRL2s in biological and biomedical research, methods that efficiently produce functional CUL2 proteins will greatly facilitate studies on the mechanism and regulation of CRL2s. Here, we report two cost-effective systems for the expression and purification of recombinant human CUL2 from *E. coli* cells. The purified CUL2 proteins were ~ 95% pure, could bind their substrate receptor modules, and were enzymatically active in transferring ubiquitin or ubiquitin-like protein to the corresponding substrate in in vitro assays. The presented methodological advancements will help advance research in CRL2 function and regulation.

## Introduction

Protein turnover is a cellular regulatory system defined by the continuous synthesis and decomposition of specific proteins to maintain the integrity of optimally functioning proteins^1,2^. Abnormalities during protein turnover, specifically during protein degradation, often result in human diseases such as cystic fibrosis and liposarcoma. A major mechanism for selective protein degradation in eukaryotes is through the ubiquitin proteasome system (UPS)^2,3^. Degradation by the UPS occurs through the covalent attachment of ubiquitin (Ub) molecules to a target protein via a process named ubiquitination. Ubiquitination is achieved by the sequential activity of three enzymes known as the E1-activating enzyme, the E2-conjugating enzyme, and the E3 ligase. The human genome encodes about 600–700 E3 ligases, among which cullin-RING ligases (CRLs) comprise the largest family of E3s^4,5^. CRLs are typically characterized by forming a horseshoe-like structure comprised of a cullin protein, a substrate receptor module that usually consists of an adaptor protein and an interchangeable substrate receptor protein, and a RING finger protein RBX1 or RBX2. The N-terminal domain (NTD) of cullin serves as a docking station for the substrate receptor module which recruits the target substrate; the C-terminal domain (CTD) of cullin binds RBX1/2 which recruits E2 loaded with Ub (E2 ~ Ub)^2,6,7^. In humans, six cullin proteins are present to serve as a scaffold for the CRL complexes^2,7^.

Cullin-2 (CUL2) is one type of cullin protein, which only exists in multi-cellular organisms^4,8^. It plays critical roles in both physiological processes such as embryogenesis, and pathogenesis of the human immunodeficiency virus (HIV-1)^4^. In the CUL2-based CRL2 complex, CUL2 serves as the platform for an adaptor protein complex, Elongin B and Elongin C (EloB/C), and the adaptor complex interacts with various substrate receptors such as the von Hippel-Lindau (VHL) tumor suppressor protein^9^. VHL targets hypoxia inducible factor 1-α (HIF1α) for ubiquitination and degradation, and misregulation of HIF1α can result in the tumorigenic VHL syndrome^10^. In recent years, co-opting cells’ proteolytic machineries for therapeutic benefit, particularly re-directing E3 ligases towards new substrates (neo-substrates) using PROteolysis TArgeting Chimeras (PROTACs), is emerging as a promising pharmacological strategy^11–14^. CRL2^VHL^ has been on the forefront of protein-degrader drug development, with numerous VHL-based PROTACs discovered in the past decade^15–32^. Because CRL2s play critical roles in protein ubiquitination and drug discovery, mechanistic insights into the activity and regulation of CRL2 will benefit the development of novel therapeutics against disease and infection.

Reconstituted in vitro assays have served as important tools in understanding enzymatic activities and specificities. Recombinant CUL2 was previously generated using a baculovirus expression system, which led to the first known structures of CUL2 in complex with RBX1, EloB/C, and VHL^33,34^. Although the baculovirus-infected insect cells can produce properly folded full-length CUL2, the production of baculovirus vectors can be time consuming, and strict, costly cell culture conditions are usually required for optimal protein yield from insect cells^35,36^. Thus, a more cost-effective method for recombinant CUL2 protein expression and purification is desirable to facilitate studies of CRL2.

Here, we report novel methods for generating human CUL2•RBX1 proteins from *E. coli* cells. With a single plasmid, the recombinant CUL2•RBX1 can be generated by either co-expressing the full-length CUL2 with RBX1 or using the “Split-n-Coexpress” strategy^35,37^. In our in vitro assays, the recombinant CUL2•RBX1 purified from either expression system can catalyze the conjugation of NEDD8 to CUL2 and can bind VHL•EloB/C to form CRL2^VHL^ that ubiquitinates the peptide substrate derived from the degron motif of HIF1α. In summary, we have established and optimized an efficient system for bacterial expression and purification of functional recombinant human CUL2•RBX1 proteins.

## Results

### Expression and purification of full-length human CUL2 from *E. coli*

**Figure 1:**
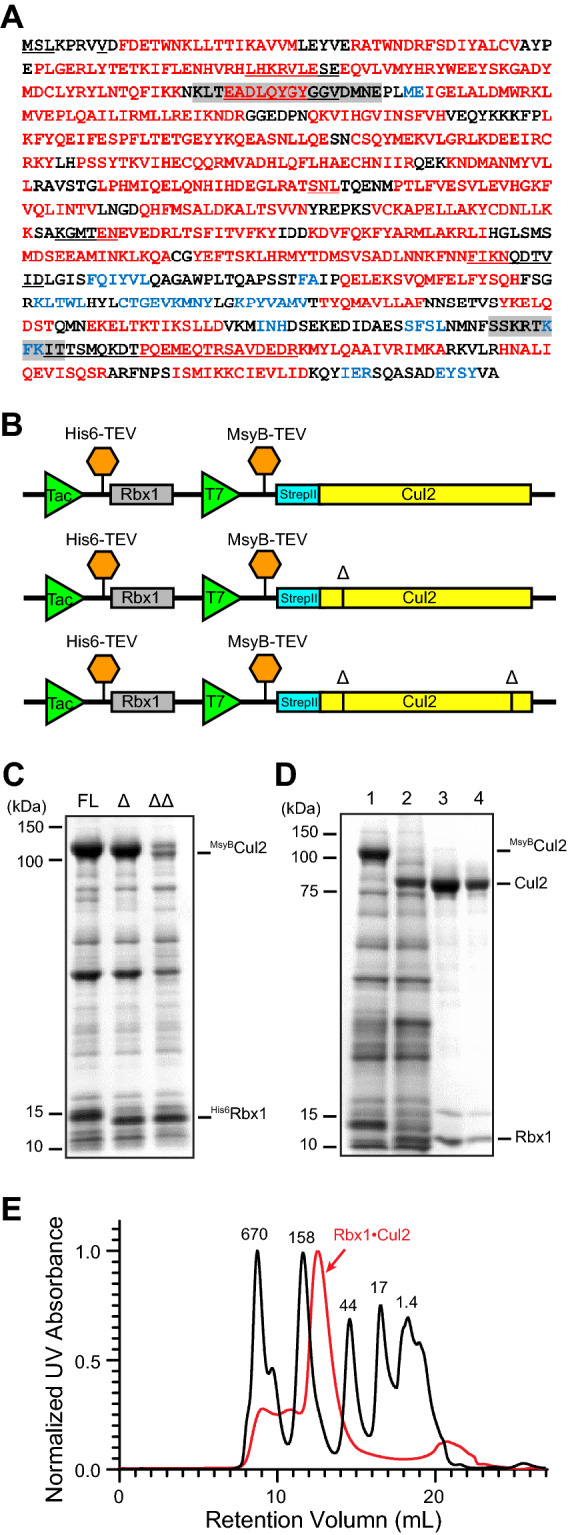
Expression of full-length RBX1•CUL2 in *E. coli* cells. (**A**) Secondary structures and disordered regions of CUL2 predicted by XtalPred. Red: helix; blue: strand; underlined: disordered region; shaded: deleted region in **B-C**. (**B**) Schematic illustrating strategies for expressing the full-length (FL) RBX1•CUL2 and its variants (RBX1•CUL2^FL^, RBX1•CUL2Δ, and RBX1•CUL2ΔΔ). Each construct contains a ^His6^RBX1 coding sequence driven by a Tac promoter and a ^MsyB^CUL2 coding sequence driven by a T7 promoter. Both His6 and MsyB can be cleaved by the TEV protease. Truncation sites (Δ) of CUL2 are shown as black lines. (**C**) RBX1•CUL2^FL^, RBX1•CUL2Δ, and RBX1•CUL2ΔΔ extracted from *E. coli* cells transformed with plasmids shown in (**B**), using Ni-NTA beads. (**D**) Expression and purification of RBX1•CUL2^FL^. Recombinant RBX1• ^StrepII^CUL2^FL^ was purified through multiple steps, including Ni-NTA affinity chromatography (#1), incubation with the TEV protease to remove the His6 tag and MsyB fusion protein (#2), Strep-tag affinity chromatography (#3), and gel filtration (#4). (**E**) Chromatogram showing gel filtration elution peaks of the protein standards and the recombinant RBX1•CUL2^FL^. Dark line: protein standards; red line: RBX1•CUL2^FL^.

Previous trials of expressing human CUL2 protein have generated the N-terminal segment of CUL2 (CUL2^1–163^) from *E. coli* cells^38^, and the full-length CUL2 in complex with RBX1 from insect cells^33,34^. The low protein solubility is a common limitation for high-level expression of full-length cullin proteins in bacteria^35^, and this limitation was overcome by deleting two short unstructured regions when full-length human CUL1•RBX1 was produced in *E. coli* ^39^. We learnt from the successful experience for CUL1•RBX1 expression and we used computational tools to predict disordered regions in the CUL2 sequence (Fig. 1A). We then found that deleting two segments in CUL2 (Fig. 1A, shaded sequences), which are invisible in the CUL2 crystal structure^33^, eliminated all disordered regions predicted in silico. Therefore, we tested if deleting these segments would yield soluble CUL2 when CUL2 was co-expressed with RBX1 in *E. coli* cells. Furthermore, because MsyB, a hyper-acidic bacterial protein, has been shown to improve protein solubility and assist correct protein folding in *E. coli* ^40^, we fused it to the N-terminus of CUL2 and placed a TEV protease cutting site after it. Key components in plasmids co-expressing ^His6^RBX1 and ^His6^MsyB-^StrepII^CUL2 are illustrated in Fig. 1B. The His6 tag and StrepII tag were added for purification purposes. The deleted segment was marked as Δ (Fig. 1B).

We co-expressed ^His6^RBX1 with the full-length (FL) ^MsyB^CUL2 (^His6^RBX1•^MsyB^CUL2^FL^), or ^MsyB^CUL2 with a segment deleted at its NTD (^His6^RBX1•^MsyB^CUL2Δ), or ^MsyB^CUL2 with segments at both NTD and CTD deleted (^His6^RBX1•^MsyB^CUL2ΔΔ). Interestingly, following affinity purification with the Ni-NTA resin, we could extract both ^His6^RBX1•^MsyB^CUL2^FL^ and ^His6^RBX1•^MsyB^CUL2Δ from *E. coli* (Fig. 1C), suggesting that the MsyB fusion protein alone improved the solubility of CUL2. Extraction of ^His6^RBX1•^MsyB^CUL2ΔΔ was not successful (Fig. 1C), likely because the deletion at CUL2 CTD disrupted the binding of RBX1 and resulted in insoluble CUL2 ^35^. The ^His6^RBX1•^MsyB^CUL2^FL^ eluted from the Ni-NTA resin was further purified through incubating with the TEV protease to remove the His6 tag and MsyB fusion protein (Fig. 1D, #2), followed by Strep-Tactin affinity chromatography (Fig. 1D, #3), and size exclusion chromatography (Fig. 1D, #4). The purified RBX1•^StrepII^CUL2^FL^ was 93% pure with a yield of ~ 0.2 mg protein per liter of culture, and importantly, its retention volume on the size exclusion chromatography suggests an apparent molecule weight (MW) of 141 kD (Fig. 1E), indicating the recombinant RBX1•^StrepII^CUL2 (estimated MW: 100 kD) was correctly structured.

### Expression and purification of human CUL2 from *E. coli* cells via “Split-n-Coexpress”

**Figure 2:**
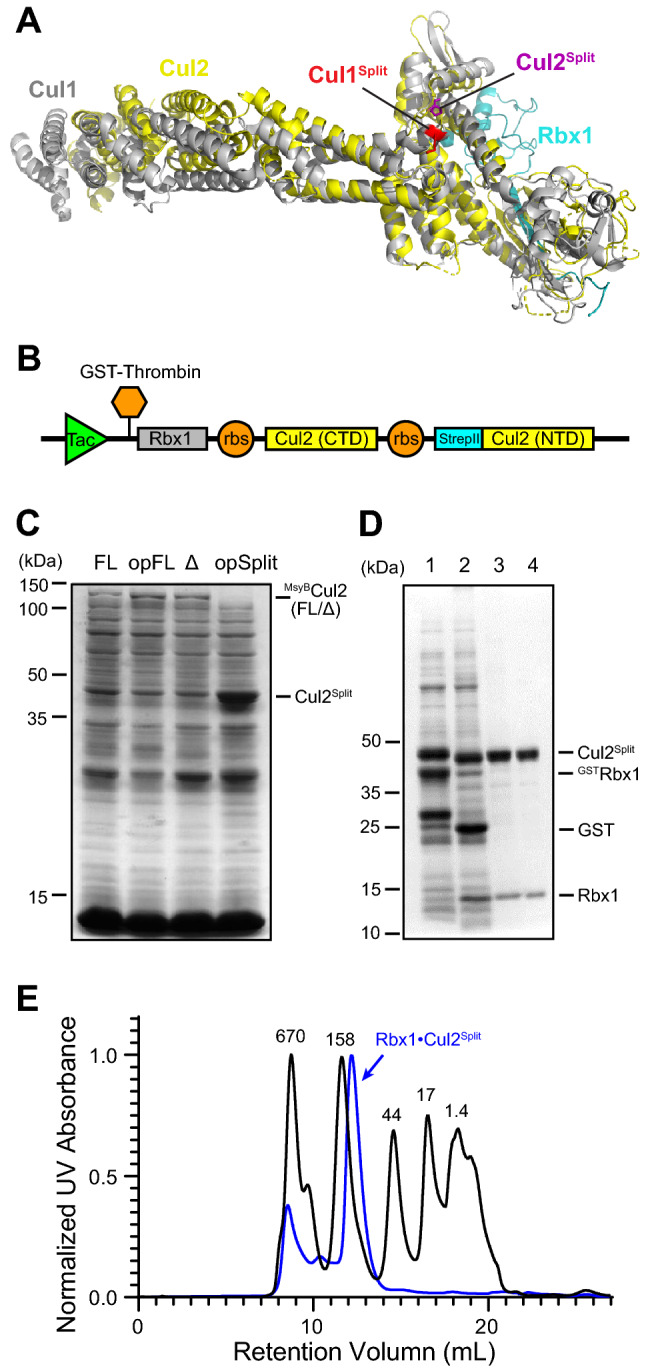
“Split-n-Coexpress” of RBX1•CUL2 in *E. coli* cells. (**A**) Structural alignment of CUL1 (PDB ID: 1U6G) and CUL2 (PDB ID: 5N4W). Splitting sites used for “Split-n-Coexpress” strategies are marked on both proteins. Grey: CUL1; yellow: CUL2; cyan: RBX1; purple: splitting site on CUL1; red: splitting site on CUL2 for expression trial. (**B**) Schematic illustrating the strategy for expressing the RBX1• ^StrepII^CUL2^Split^ via “Split-n-Coexpress”. The construct contains coding sequences of ^GST^RBX1 and the two halves of CUL2 (CTD, NTD) that are linked by ribosomal binding sites (RBS) and is driven by a Tac promoter. Tags and fusion proteins can be cleaved by the thrombin protease. (**C**) Comparison among different methods for expressing RBX1•CUL2 in *E. coli* cells. Excess amount of Strep-Tactin resin was used to extract ^StrepII^CUL2 expressed via different strategies. FL: RBX1• ^StrepII^CUL2^FL^; Δ: RBX1• ^StrepII^CUL2Δ as in Fig. 1; opFL: RBX1• ^StrepII^CUL2^FL^ with CUL2 coding sequence codon optimized; opSplit: RBX1• ^StrepII^CUL2^Split^ with the CUL2 coding sequence codon optimized. (**D**) Expression and purification of RBX1•CUL2^Split^. Recombinant RBX1• ^StrepII^CUL2^Split^ was purified through multiple steps, including GST affinity chromatography (#1), incubation with the thrombin protease to remove the His6 tag and GST fusion protein (#2), Strep-tag affinity chromatography (#3), and gel filtration (#4). (**E**) Chromatogram showing gel filtration elution peaks of the protein standards and the recombinant RBX1•CUL2^Split^. Dark line: protein standards; blue line: RBX1•CUL2^Split^.

After successful expression and purification of RBX1•CUL2^FL^, we then sought to improve the yield of the recombinant protein. Besides deleting the disordered region in the CUL2 NTD (CUL2Δ), we also tried codon optimization of the CUL2 coding sequence and the “Split-n-Coexpress” strategy. “Split-n-Coexpress”, which splits the cullin protein into halves and co-expresses them with RBX1, was firstly reported for expressing RBX1•CUL1 in *E. coli* cells^35^. In search of a way to split CUL2, we aligned crystal structures of CUL1 and CUL2 (Fig. 2A), and we found that the counterpart for the CUL1 splitting site is after the 14th helix in CUL2 (Fig. 2A). Thus, we generated a construct to co-express ^GST^RBX1, ^StrepII^CUL2^2–380^, and CUL2^381–745^ (Fig. 2B). We then used excess amounts of Strep-Tactin resin to extract different variants of RBX1•^StrepII^CUL2 expressed in *E. coli*, including RBX1•^StrepII^CUL2^FL^, RBX1•^StrepII^CUL2Δ, RBX1•^StrepII^CUL2^opFL^ with CUL2 codon optimized, and RBX1•^StrepII^CUL2^opSplit^ with CUL2 split and codon optimized. The results showed that while deleting the disordered segment at NTD or codon optimization alone increased the amount of extractable ^StrepII^CUL2, “Split-n-Coexpress” offered a significant improvement (Fig. 2C). We thus expressed and purified the RBX1•^StrepII^CUL2^Split^ through glutathione affinity chromatography (Fig. 2D, #1), thrombin cleavage (Fig. 2D, #2), Strep-Tactin affinity chromatography (Fig. 2D, #3), and size exclusion chromatography (Fig. 2D, #4). We obtained RBX1•^StrepII^CUL2^Split^ that was over 98% pure with a yield of ~ 0.3 mg protein per liter of culture, and its retention volume on the size exclusion chromatography (Fig. 2E) was almost the same as for RBX1•^StrepII^CUL2^FL^ (Fig. 1E).

### Activities of the recombinant RBX1•CUL2 purified from *E. coli*

**Figure 3:**
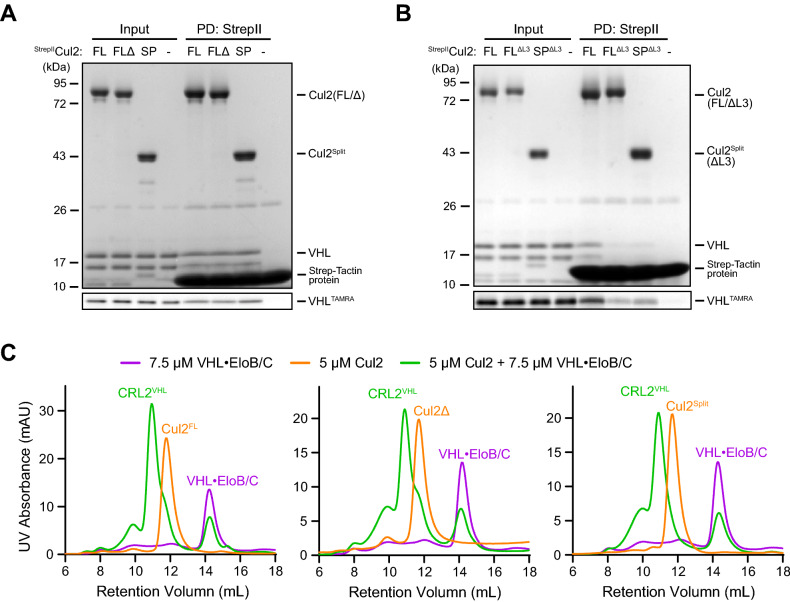
Interactions between the purified RBX1•CUL2 proteins and VHL•EloB/C. (**A-B**) StrepII-tag affinity pulldown assays for the formation of CRL2^VHL^ complex when different types of RBX1•CUL2 is present. In each assay, 2 µM VHL•EloB/C or VHL^TAMRA^•EloB/C was incubated with 1 µM each type of RBX1•CUL2. The input and pulldown samples were fractionated on SDS-PAGE, scanned for TAMRA signal, and stained with Coomassie Blue. FL: RBX1• ^StrepII^CUL2^FL^; FLΔ: RBX1• ^StrepII^CUL2Δ; SP: RBX1•^StrepII^CUL2^Split^; ΔL3: Leucine 3 of CUL2 deleted. (**C**) Chromatograms showing gel filtration elution peaks of 7.5 µM VHL•EloB/C (purple), 5 µM indicated version of RBX1•CUL2 (orange), and mixtures of 7.5 µM VHL•EloB/C and 5 µM RBX1•CUL2 (green). The same trace for VHL•EloB/C was used in all three plots. Based on the reduction of the VHL•EloB/C peak area, percentage of RBX1•CUL2 assembled into CRL2^VHL^ complex was estimated as 79% for CUL2^FL^, 82% for CUL2Δ, and 83% for CUL2^Split^. Protein species under the main peaks were confirmed by SDS-PAGE (Fig. S1).

To determine if the recombinant RBX1•CUL2 purified from *E. coli* cells retained their activity, we first tested the binding of VHL•EloB/C, a substrate receptor module for CUL2, with our recombinant RBX1•CUL2. Our pulldown assay with the StrepII tag showed that RBX1•^StrepII^CUL2^FL^, RBX1•^StrepII^CUL2Δ, and RBX1•^StrepII^CUL2^Split^ all bound to VHL•EloB/C at similar degrees (Fig. 3A). Furthermore, previous studies have shown that the third amino acid in CUL2, a Leucine (L3), is important for EloC binding^38^. Thus, we deleted L3 from our CUL2^FL^ (FL^ΔL3^) and CUL2^Split^ (SP^ΔL3^) protein, and consistent with the previous report, we found that the loss of L3 significantly reduced the binding of CUL2 to VHL•EloB/C (Fig. 3B). Furthermore, to access what stoichiometric proportion of the RBX1•CUL2 could bind VHL•EloB/C, we mixed 5 µM each version of RBX1•CUL2 with 7.5 µM VHL•EloB/C and analyzed the protein mixture on size exclusion chromatography, similarly to that performed before^33,41^. As shown in Fig. 3C, the protein mixture was eluted as two major peaks (green lines). The first peak was eluted earlier than RBX1•CUL2 (orange lines), and it contained both RBX1•CUL2 and VHL•EloB/C (Fig. S1), suggesting the formation of the pentameric CRL2^VHL^ complex. The second peak contained VHL•EloB/C (Fig. S1), and it showed the same retention volume as VHL•EloB/C (purple lines) with a reduced peak area. Because peak areas represent amounts of the detected analyte, by quantifying the reduction in the VHL•EloB/C peak area, we estimated that 79%-83% of RBX1•CUL2 in the protein mixture was in complex with VHL•EloB/C (Fig. 3C). Taken together, these results demonstrated that our recombinant RBX1•CUL2 can efficiently recruit the substrate receptor module to form a CRL2 complex.

**Figure 4:**
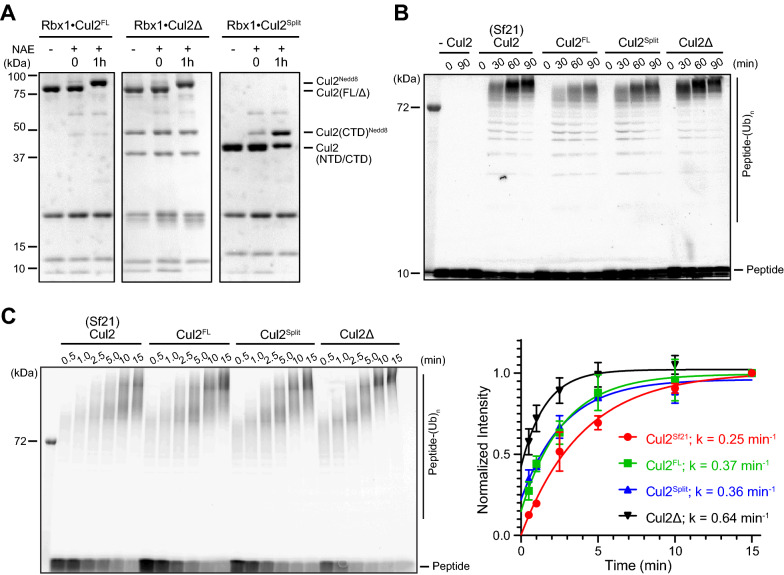
The enzyme activity of the recombinant RBX1•CUL2 proteins. (**A**) The purified CUL2^FL^, CUL2Δ, and CUL2^Split^ can be efficiently neddylated. Protein samples with indicated reaction conditions were fractionated on SDS-PAGE and stained with Coomassie Blue. (**B**) The purified RBX1•CUL1^FL^, RBX1•CUL2Δ, and RBX1•CUL2^Split^ can efficiently ubiquitinate a peptide substrate derived from the degron motif of HIF1α. The peptide substrate contains a TAMRA fluorescent dye. Recombinant RBX1•CUL2 produced from insect cells (Sf21) was included as a positive control, whereas samples with no CUL2 were included as a negative control. Protein samples with indicated reaction conditions were fractionated on a 16% SDS-PAGE gel and scanned for TAMRA signal to detect the unmodified and ubiquitinated peptide substrate. (**C**) Quantitative comparison of CRL2^VHL^ ubiquitination activities. The same assay as in (**B**) was performed with indicated time points, and samples were fractionated on 4–20% SDS-PAGE gels. TAMRA signal intensities of ubiquitinated substrates were quantified, normalized to signals of the last sample points, and averaged. Single exponential fits with the normalized intensities were shown in the plot on the right, together with k values from the regression for each RBX1•CUL2 variant. Error bar: standard error (n = 3).

We then tested if the recombinant RBX1•CUL2 was enzymatically active. Conjugating the small protein NEDD8 to cullin is one of the key mechanisms for CRL activation^2,42,43^. This process, referred to as neddylation, is achieved by recruiting E2 ~ NEDD8 via RBX1 and subsequently transferring NEDD8 to cullin CTD^2,42,43^. We performed in vitro neddylation of our recombinant RBX1•CUL2, including RBX1•CUL2^FL^, RBX1•CUL2Δ, and RBX1•CUL2^Split^. We found that the protein bands representing CUL2^FL^, CUL2Δ, or CUL2^381–745^ (CUL2^CTD^) shifted upwards on the SDS-PAGE gel after incubating with the neddylation enzymes (Fig. 4A), suggesting that each variant of CUL2 was fully conjugated to NEDD8. We further tested if the neddylated RBX1•CUL2, once associated with VHL•EloB/C, can ubiquitinate its substrate, a peptide derived from the amino-terminal oxygen-dependent degradation (NODD) motif of HIF1α^44^. This peptide substrate, which differs from the CODD degron sequence characterized previously^33,44,45^, contains a hydroxylated Proline (P402) that is required for VHL binding^46^, a Lysine receptor (K389) for Ub^47^, and a TAMRA fluorophore at the C-terminus for detection. Besides the three variants of RBX1•CUL2 we purified from *E. coli*, we also included RBX1•CUL2 purified from insect cells as a positive control, and a no RBX1•CUL2 mixture as a negative control. As shown in Fig. 4B, fluorescence scan for TAMRA signal revealed that when no RBX1•CUL2 was present, the peptide substrate was unchanged. In contrast, when each type of RBX1•CUL2 was present, TAMRA labeled molecules with higher molecular weights appeared and accumulated over time. To further access if the enzymatic activities differ among different versions of RBX1•CUL2, we repeated the substrate ubiquitination assay with more time points and shorter time periods (Fig. 4C, left panel). By comparing the rates at which ubiquitinated substrates were generated, we found that RBX1•CUL2 purified from *E. coli* cells displayed activities similar to, or slightly greater than, RBX1•CUL2 purified from insect cells (Fig. 4C, right panel). Based on these results, we conclude that all forms of the recombinant RBX1•CUL2 we purified from *E. coli* cells were enzymatically active.

## Discussion

We have developed new cost-effective systems to generate recombinant human RBX1•CUL2. We found that the addition of an MsyB fusion protein helped producing soluble full-length CUL2 in complex with RBX1 from bacterial cells. This is likely because the MsyB protein assists protein folding specifically in *E. coli* cells by reducing aggregation of the targeted protein^40^. In addition to the MsyB fusion protein, we also found that deleting one unstructured region at the CUL2 NTD (RBX1•CUL2Δ) increased the yield of soluble protein.

After successfully producing the full-length CUL2 protein in *E. coli*, we further increased our protein yield with codon optimization of the CUL2 coding sequence and the “Split-n-Coexpress” strategy. In the “Split-n-Coexpress” system, the cullin protein is divided into two halves, which allows for the full expression of two smaller subunits. When folding, the two subunits dock into each other at a hydrophobic interface to assemble the functional full protein^35,37^. With the structural alignment of CUL1 and CUL2, we determined the split site for CUL2, and we successfully produced RBX1•CUL2^Split^ via the “Split-n-Coexpress” system. We found that both codon optimization and “Split-n-Coexpress” were beneficial to our protein production, while the “Split-n-Coexpress” offered a greater improvement.

After purifying our RBX1•CUL2^FL^, RBX1•CUL2Δ, and RBX1•CUL2^Split^ proteins with two affinity chromatography, site-specific protease cleavage for tag removal, and size exclusion chromatography, we then tested their biochemical activities. We first demonstrated that all versions of the recombinant RBX1•CUL2 could efficiently form CRL2 through recruiting its substrate receptor module VHL•EloB/C, unless the L3 at CUL2 NTD was intentionally deleted. This result is consistent with the previous finding that the L3 of Cul2 is important for the binding of EloC^38^, and it shows that our recombinant RBX1•CUL2 can detect changes in the affinity of CRL2. We then showed that all forms of our recombinant RBX1•CUL2 could be fully conjugated to NEDD8, a key modification of cullin that promotes CRL activities^2,4,43,48^. Lastly and importantly, we confirmed that our purified RBX1•CUL2 proteins were capable of ubiquitinating its substrate. Because HIF1α is a well-studied CUL2 substrate^4,49^, we used a hydroxylated peptide that corresponds to a degron motif of HIF1α as the substrate for our RBX1•CUL2 in the in vitro ubiquitination assay. As a positive control, we also included full-length RBX1•CUL2 produced from insect cells, which was used in previous structural studies of CRL2 ^33,34^. We showed that in the presence of RBX1•CUL2 produced from either bacteria or insect cells, the peptide substrates were ubiquitinated over time and increasing amounts of higher molecular weight products were detected. Of note, we found that when mixed with slight excess amount of VHL•EloB/C, around 80% of bacteria produced RBX1•CUL2 could form the CRL2^VHL^ complex. While this percentage of complex assembly is lower than RBX1•CUL2 purified from insect cells^33,41^, all forms of bacteria produced RBX1•CUL2 showed ubiquitination activities equivalent to RBX1•CUL2 produced from insect cells.

In conclusion, our study provides novel and efficient methods for producing fully functional recombinant RBX1•CUL2 in *E. coli*. We expect that the new expression systems we established will facilitate future research on CRL2, such as structural or mechanistic studies of factors/mutations that alter CRL2 assembly or activity. Further, in vitro ubiquitination assays similar to what we reported here will help validate novel natural or neo-substrates of CRL2s, study kinetics of substrate ubiquitination, as well as characterize regulators or PROTACs that modulate the enzymatic activity of CRL2s.

## Methods

### Constructs

For generating the construct co-expressing RBX1 and CUL2, a pGEX vector (Sigma-Aldrich) was firstly edited using the Q5 Site-Directed Mutagenesis Kit (NEB BioLabs), to replace the GST coding sequence with DNA sequence encoding a His6 tag followed by a TEV protease cutting site (ENLYFQS) and a few restriction enzyme cutting sites. The following DNA fragments were sequentially inserted after the TEV site: RBX1 coding sequence (with NdeI/NotI sites), T7 promoter (with NotI/NcoI sites), MsyB coding sequence followed by a TEV site (with NcoI/NheI sites), and ^StrepII^CUL2 coding sequence (with NheI/SalI sites). Codon optimized CUL2 coding sequence was synthesized (Gene Universal) and inserted with NheI/SalI sites. Loop deletion(s) of CUL2 were introduced using the Q5 Site-Directed Mutagenesis Kit (NEB BioLabs). The construct expressing RBX1•^StrepII^CUL2^Split^ was generated through modifying the pCool vector (Addgene Plasmid #29,519, a gift from Ning Zheng). First, the CUL1 coding sequence was replaced by DNA sequence encoding codon optimized CUL2^381–745^ (with NcoI/NotI sites). Then the sequence of codon optimized ^StrepII^CUL2^2–380^ with the RBS sequence preceding it was inserted via the NotI site. Sequences of all the constructs were confirmed by Sanger sequencing.

### Expression and purification of RBX1•CUL2

Different versions of RBX1•^StrepII^CUL2 were expressed in BL21 (DE3) *E. coli* with 0.2 mM IPTG induction overnight at 16°C^50,51^. The bacterial cells were pelleted and lysed via sonication in buffer containing 30 mM Tris-HCl (pH 7.6), 200 mM NaCl, 10% glycerol, 5 mM DTT, 1 × Protease Inhibitor Cocktail (PIC, Roche)^50,51^. The full-length RBX1•^StrepII^CUL2 was purified on Ni-NTA resin (Roche) followed by digestion with TEV protease. The RBX1•^StrepII^CUL2^Split^ was purified on glutathione resin (Cytiva) followed by digestion with thrombin (Sigma-Aldrich). After the protease digestion was complete, the protein sample was passed through a Strep-Tactin Superflow cartridge (IBA Lifesciences) on an ÄKTA pure chromatography system (Cytiva), washed with Buffer W [100 mM Tris-HCl (pH 8.0), 150 mM NaCl], and the StrepII tagged protein was eluted with Biotin Elution Buffer [100 mM Tris-HCl (pH 8.0), 300 mM NaCl, 5% glycerol, 50 mM biotin]. The eluate was then concentrated using a centrifugal filter unit (50 kD cutoff, MilliporeSigma), and purified through a S200 size exclusion column (Cytiva) on the ÄKTA system. The Gel Filtration Standard (Bio-Rad) protein mixture was passed through the same S200 size exclusion column with the same condition in a separate run. Purified RBX1•^StrepII^CUL2 were aliquoted and stored in Storage Buffer [30 mM Tris-HCl (pH 7.5), 100 mM NaCl, 10% glycerol, 1 mM DTT] at -80°C^50,51^. RBX1•CUL2 purified from Sf21 insect cells was purchased from R&D Systems.

### Expression and purification of VHL•EloB/C

VHL^54–213^•EloB/C complex was expressed by co-transforming BL21 (DE3) *E. coli* with XLB250 and XLB192 and inducing overnight at 16°C^50,51^. It was then purified on glutathione resin followed by on-column digestion with thrombin overnight at 4 °C. Protein released from the glutathione resin to the supernatant was concentrated via a centrifugal filter unit (10 kD cutoff, MilliporeSigma) and purified through a S75 size exclusion column (Cytiva) on the ÄKTA system. Purified VHL•EloB/C were aliquoted and stored in Storage Buffer at -80 °C.

### In vitro neddylation and ubiquitination assay

CUL2 neddylation reactions were performed for 1 h at room temperature in Reaction Buffer [30 mM Tris (pH 7.5), 5 mM MgCl_2_, 2 mM DTT, 2 mM ATP] containing 0.25 μM NAE, 3 μM UBC12, 2 μM NEDD8 (BostonBiochem), and 1 μM different versions of recombinant RBX1•CUL2 ^52^. Reaction mixtures with or without the 1-h incubation period were mixed with 4 × SDS sample buffer, fractionated with SDS-PAGE, and visualized through Coomassie Blue staining. In vitro ubiquitination was performed at room temperature in Reaction Buffer containing 0.2 μM different versions of RBX1•CUL2, 0.2 μM VHL•EloB/C, 1 μM Ub-E1, 2 μM Cdc34, 60 μM ubiquitin, and 0.8 μM HIF1α degron peptide [Ac-KLRREPDALTLLA-(hydroxylated-P)-AAGDTIISLDFGSNGRRASYK(TAMRA)-amide] (synthesized by Biomatik). At indicated time points, aliquots of the reaction mixtures were withdrawn and mixed with 4 × SDS sample buffer. Samples were fractionated with SDS-PAGE and analyzed by a Typhoon scanner (Cytiva) to detect the TAMRA signal from the degron peptide. To compare ubiquitination rates, signals of ubiquitinated peptides were quantified using ImageJ, and the intensity from each sample was normalized to the last time point (15 m) of the same set of samples. The normalized intensities from three replicates were then averaged and fit to single exponential curves in Prism 8 (GraphPad) with no constraint applied. The k values were calculated by Prism 8 after the curve fitting. Concentrations of RBX1•CUL2 protein stocks were calibrated by Coomassie blue stain and Western blot prior to the assay to ensure equal amounts of RBX1•CUL2 were present in all reactions.

### StrepII pull-down assay

Different versions of purified RBX1•^StrepII^CUL2 (1 μM) was individually mixed with 2 μM VHL•EloB/C (or 2 μM VHL^TAMRA^•EloB/C) in Storage Buffer, and 100 μL of the protein mixture was incubated with 30 μL of Strep-Tactin resin slurry (IBA Lifesciences) for 30 min at room temperature. The resin was then washed with Storage Buffer and the bound proteins were eluted using 30 μL of 2 × SDS sample buffer. Both the sample input and protein eluate were fractionated with SDS-PAGE and visualized through Coomassie Blue staining. The negative control was prepared and analyzed in the same way except that no RBX1•^StrepII^CUL2 was added.

## Supplementary Information

Supplementary Information.
