# Supplementary material for: Expression and purification of functional recombinant CUL2•RBX1 from E. coli
Source: Sci Rep. 2021 May 27;11:11224. doi: 10.1038/s41598-021-90770-x (PMC8160325; doi:10.1038/s41598-021-90770-x)
Supplement: Supplementary file 1 — Supplementary Information. [file 41598_2021_90770_MOESM1_ESM.pdf]

Expression and purification of functional recombinant CUL2•RBX1 from *E. coli*

Stephanie Diaz[1], Lihong Li[1,2], Kankan Wang[1], Xing Liu[1, 2, *]

[1]Department of Biochemistry, Purdue University, West Lafayette, Indiana, United States

[2]Center for Plant Biology, Purdue University, West Lafayette, Indiana, United States

[*]Correspondence and requests for materials should be addressed to X.L.

Email: xingliu@purdue.edu

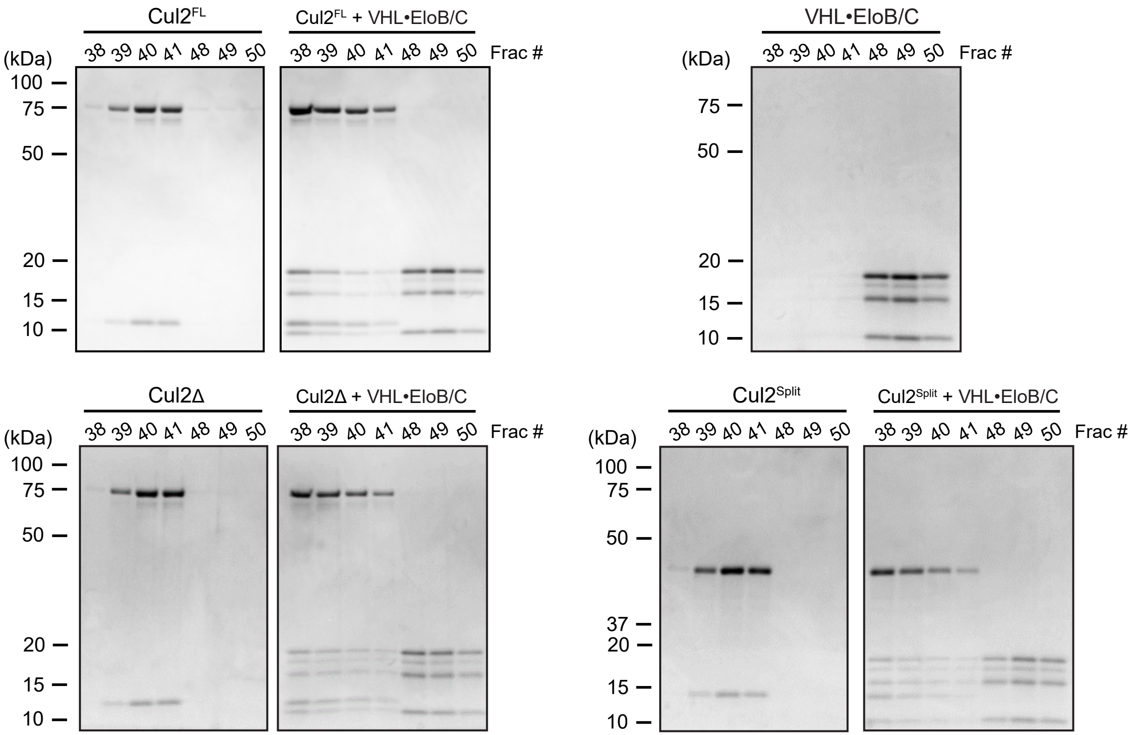

Fig. S1 Confirmation of protein species in gel filtration fractions. Related to Fig. 3C. Samples collected from the indicated fractions eluted from a Superdex 200 Increase 10/300 GL column (Cytiva) were fractionated on 4-20% gradient SDS-PAGE gels and stained with Coomassie blue. Protein samples injected to the column are indicated above each gel. The eluate was collected as 300 μL fractions.

Figure 1C

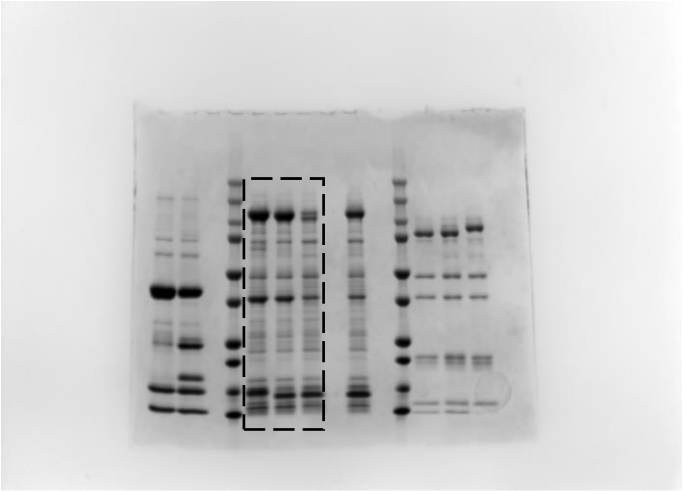

Figure 1D

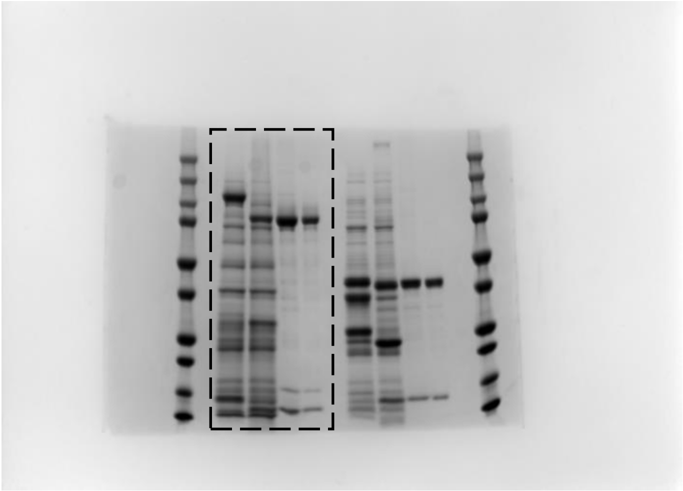

Fig. S2 Uncropped gel images for Fig. 1

Figure 2C

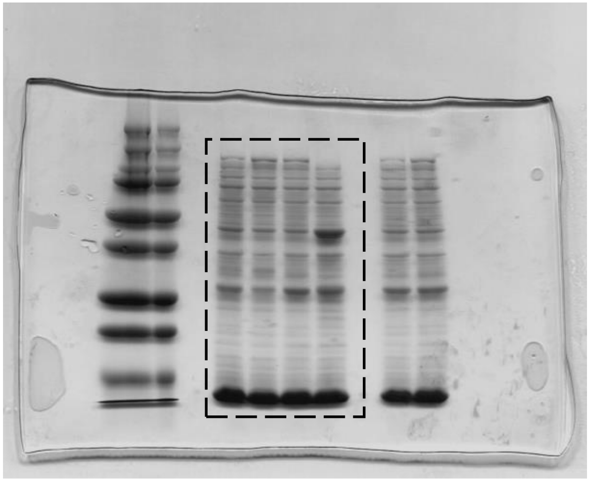

Figure 2D

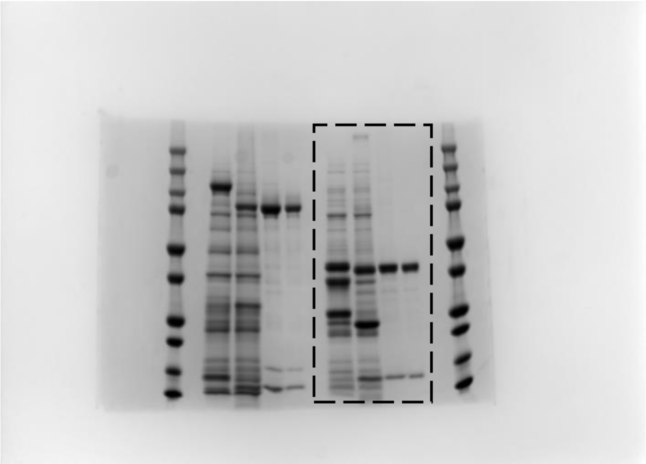

Fig. S3 Uncropped gel images for Fig. 2

Figure 3A

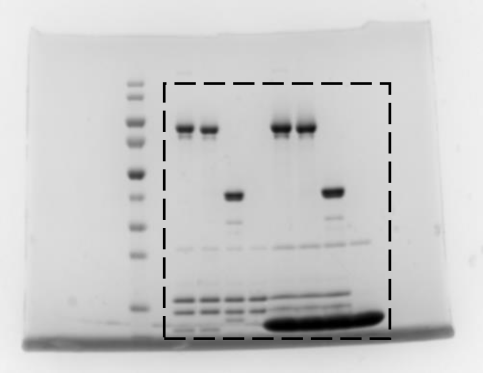

Figure 3A

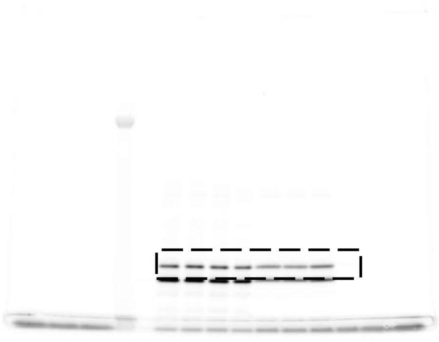

Figure 3A
(alternative exposure)

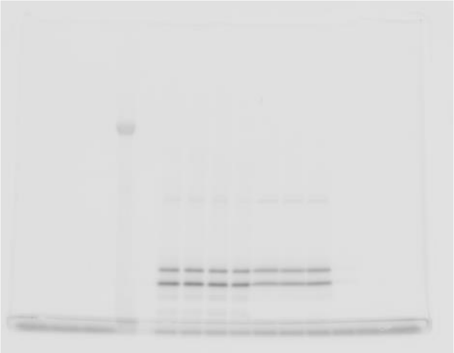

Figure 3B

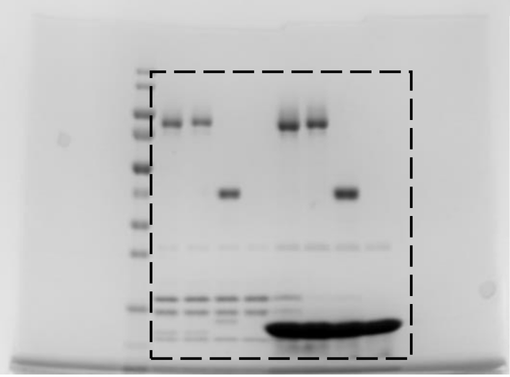

Figure 3B

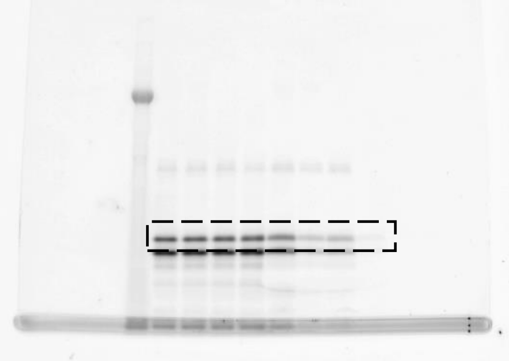

Figure 3B
(alternative exposure)

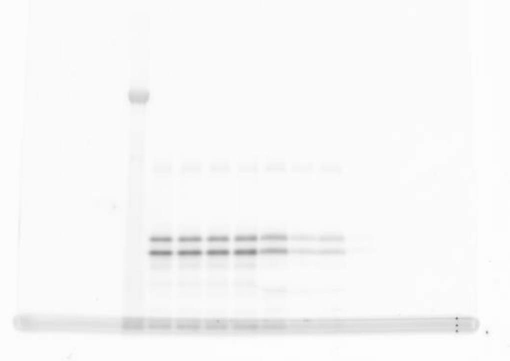

Fig. S4 Uncropped gel images for Fig. 3, including alternative exposure for 3A and 3B.

Figure 4A

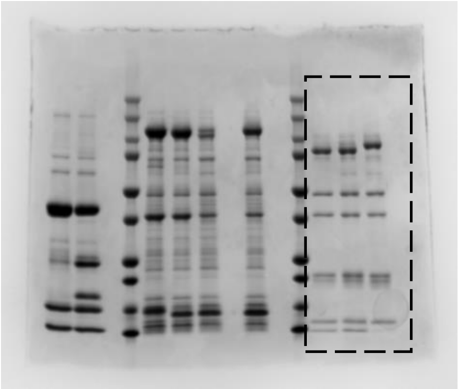

Figure 4A

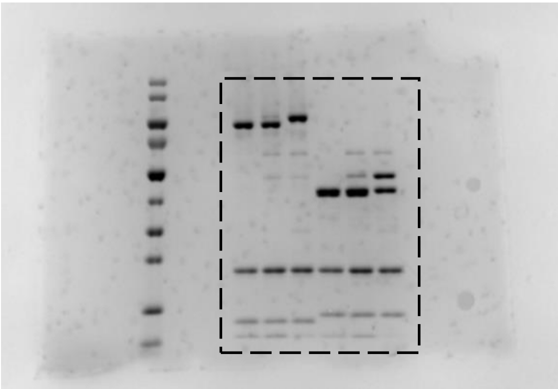

Figure 4B

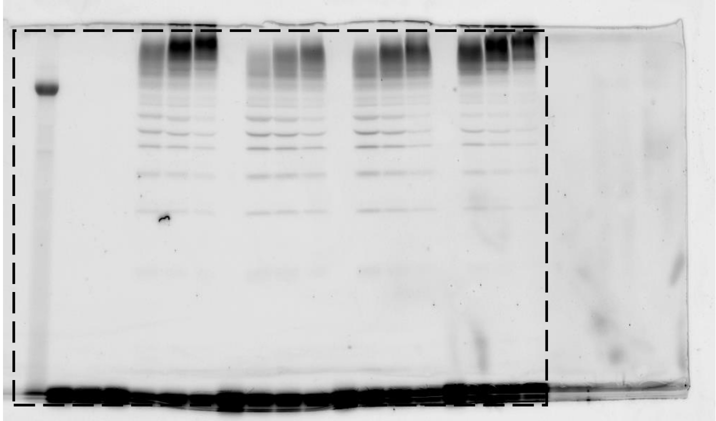

Figure 4C

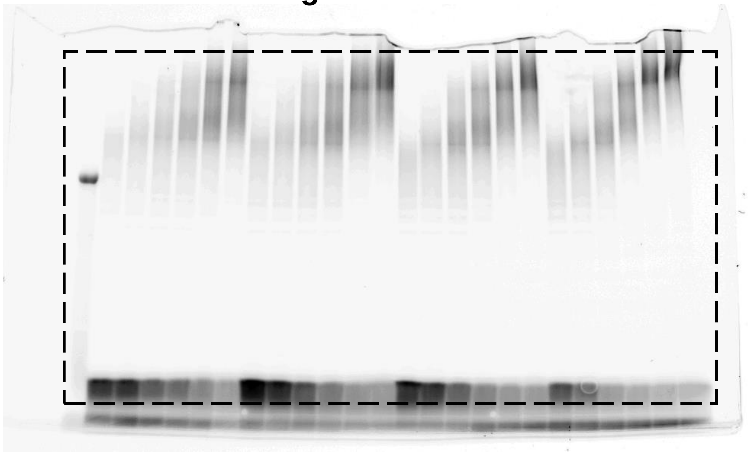

Fig. S5 Uncropped gel images for Fig. 4

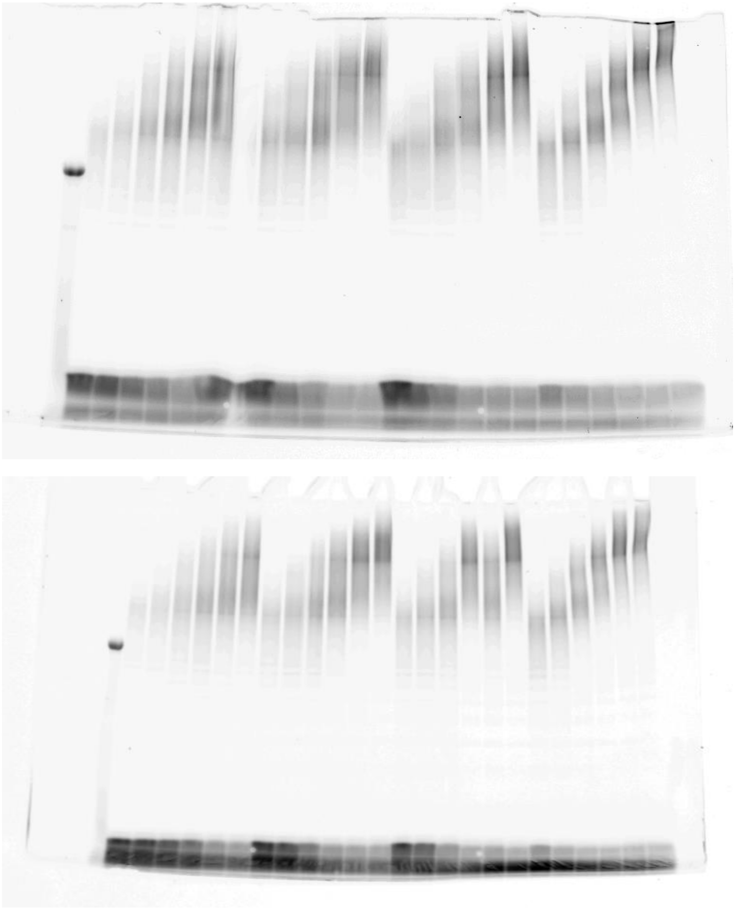

Fig. S6 Uncropped gel images for replicated experiments used in Fig. 4C

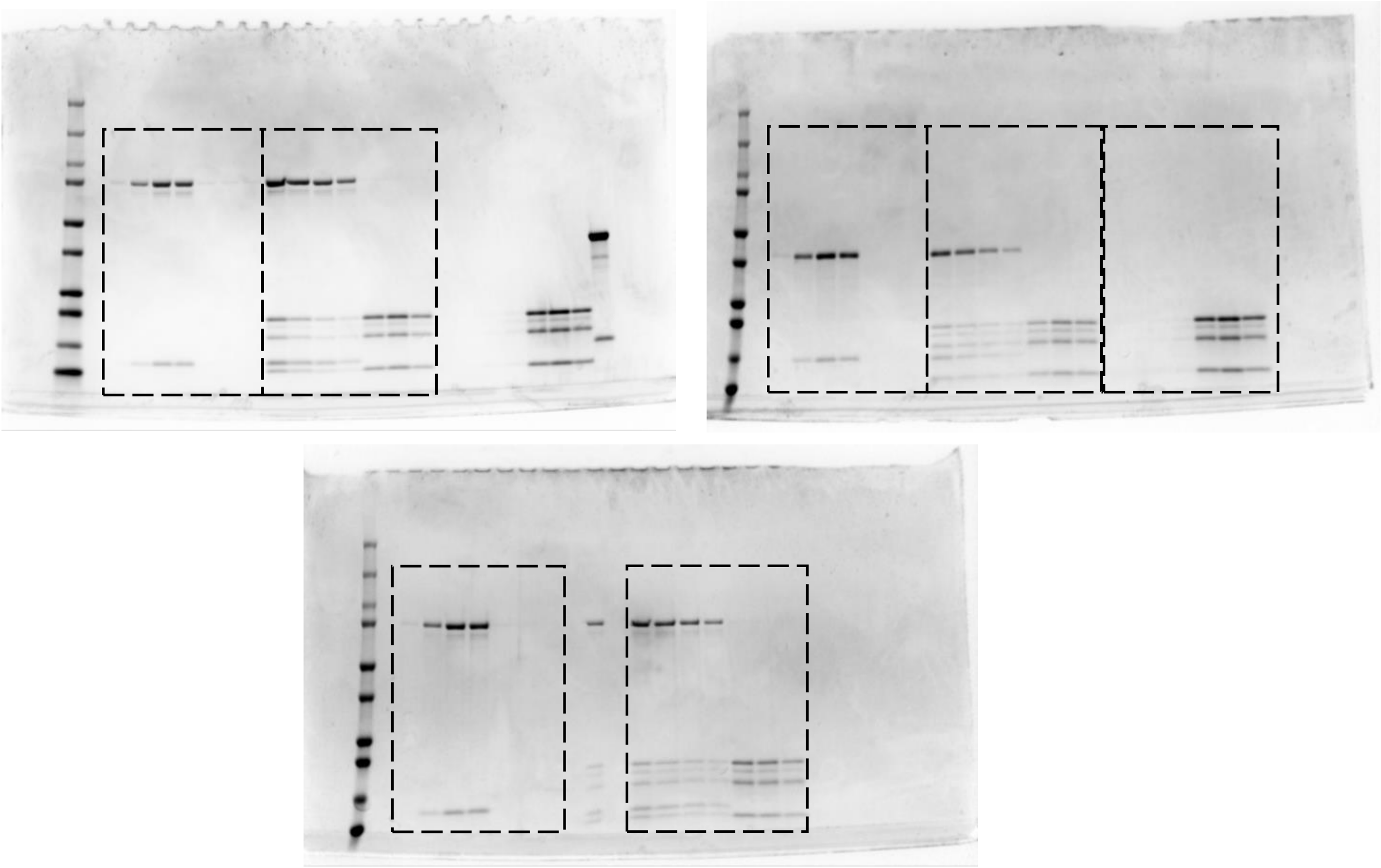

Fig. S7 Uncropped gel images for Fig. S1
